# Neural functions vary by return-to-sport status in participants with anterior cruciate ligament reconstruction: a retrospective cohort study using sub-bands of resting-state functional magnetic resonance

**DOI:** 10.3389/fnhum.2024.1457823

**Published:** 2024-11-01

**Authors:** Hongyun Song, Sunan Zhu, Zongyou Pan, XiaoJing Yu, Bing Xiong, Xuesong Dai

**Affiliations:** ^1^Department of Orthopedic Surgery, The Second Affiliated Hospital, Zhejiang University School of Medicine, Hangzhou, China; ^2^Orthopedics Research Institute of Zhejiang University, Hangzhou, China; ^3^Key Laboratory of Motor System Disease Research and Precision Therapy of Zhejiang Province, Hangzhou, China; ^4^Clinical Research Center of Motor System Disease of Zhejiang Province, Hangzhou, China; ^5^Department of Rehabilitation, The Second Affiliated Hospital, Zhejiang University School of Medicine, Hangzhou, China

**Keywords:** anterior cruciate ligament reconstruction, functional magnetic resonance, amplitude of low-frequency fluctuations, regional homogeneity, return to sports

## Abstract

**Objective:**

This study aimed to characterize the differences in neural function among patients with different functional abilities 2 years after anterior cruciate ligament reconstruction (ACLR).

**Design:**

Resting-state functional magnetic resonance imaging was performed to obtain blood-oxygen-level-dependent values for ACLR returned to sports coper participants (CP), non-coper participants (NP), and healthy controls (HC). The amplitude of low-frequency fluctuations (ALFF) and regional homogeneity (ReHo) calculated changes in the standard frequency band (SFB) (0.01–0.08 Hz), Slow4 (0.027–0.073 Hz), and Slow5 (0.01–0.027 Hz). Clinical correlations were investigated.

**Results:**

The right cerebellum_8 and bilateral putamen in SFB, while the right cerebellum_crus2 and left putamen in Slow5 were higher in CP than in NP. The ALLF values of the bilateral putamen in Slow4 were increased, while the right parietal lobule in Slow4 and left upper temporal pole in Slow5 were lower in CP than in HC. The ReHo values in the CP group in the right cerebellum_crus2 was higher than that in the NP group in Slow5 (voxel *p* < 0.05, cluster *p* < 0.05, Gaussian Random Field theory correction). Y-balance test was correlated with cerebellum ALFF values; Tegner was moderately correlated with putamen ALFF values (*p* < 0.05). Knee Injury and Osteoarthritis Outcome Score-sports, International Knee Documentation Committee Subjective Knee Evaluation Form and Tegner scores were correlated with the ReHo values of right cerebellum_crus2 (*p* < 0.05).

**Conclusion:**

Subcortical function transfer was performed in patients with ACLR who returned to sports postoperatively: the function of the somatosensory brain area decreased, while that of the subcortical cerebellum and basal ganglia and cerebellum ReHo increased in CP, which was correlated with clinical function. ALFF and ReHo are consistent to some extent, and sub-band studies can reveal information on different brain functions compared to the classical band.

## 1 Introduction

Anterior cruciate ligament (ACL) injuries are one of the most common sports injuries worldwide (Ardern et al., 2014). Even after anterior cruciate ligament reconstruction (ACLR) and postoperative rehabilitation, the incidence of reinjury and risk of contralateral ACL tears remain high (Paterno et al., 2014). Studies have suggested that neuroplastic changes due to lost mechanoreceptors of the ACL and compensations in neuromuscular control and brain function remodeling occur after ACL injury (An et al., 2018; Baumeister et al., 2011; Baumeister et al., 2008; Luc-Harkey et al., 2017; Pietrosimone et al., 2015). Resting-state functional magnetic resonance imaging (rs-fMRI), with its noninvasive nature, high spatiotemporal resolution, and easy localization, can help analyze the function of the living human brain (Fox and Raichle, 2007). Amplitude of low-frequency fluctuations (ALFF) can calculate the low-frequency amplitude of the brain voxel level (Zang et al., 2007). The fractional amplitude of low-frequency fluctuations (fALFF) is the ALFF of a given frequency band expressed as a fraction of the sum amplitudes across the whole frequency range (Zou et al., 2008). While both measures exhibited moderate to high test–retest reliability within gray matter regions, ALFF is more reliable than fALFF in gray matter regions, making it potentially more sensitive for discerning differences between individuals and groups (Zuo et al., 2010). However, it can be impaired and influenced by non-neural physiological fluctuations, such as respiration, cardiac action, and motion (Zuo et al., 2010). Compared with ALFF, fALFF can reduce the influence of physiological noise in the ventricles and cisterns on the results (Zou et al., 2008) and reflect gray matter signals with more specificity. However, it is less reliable than ALFF as a proportional measure (Zuo et al., 2010). Regional homogeneity (ReHo) is an analytical method of rs-fMRI that measures the similarity of a given voxel’s time series to its nearest neighbors by calculating the Kendall coefficient, which is used to detect subtle changes in the synchronization of neuronal activity in a specific brain region (Zang et al., 2004).

Most studies on the ALFF of rs-fMRI have focused on low-frequency oscillatory signals in the standard frequency band (SFB) (0.01–0.08 Hz). However, many studies have revealed that some brain regions have different sensitivities to oscillatory signals in various frequency bands, resulting in the lack of certain neural activity information. Therefore, Zuo et al. (2010) and Buzsáki and Draguhn (2004) divided the ALFF into multiple frequency bands and considered Slow4 (0.027–0.073 Hz) and Slow5 (0.01–0.027 Hz) better reflectors of neuronal function, and ALFF in Slow4 (0.027–0.073 Hz) band were most robust in the basal ganglia (Zuo et al., 2010). There is a lack of sub-band research on the postoperative ACLR.

To the best of our knowledge, the ALFF and fALFF calculation were applied to the early postoperative study of ACLR (Wang et al., 2019), but not to the participants who returned to sports 2 years postoperatively. ALFF with its more stable signal could reflect individual differences, and the ReHo study was not applied to the postoperative study of ACLR. Therefore, this study aimed to explore the differences in neural plasticity in sub-band ALFF and ReHo values among patients who returned to sports (coper, CP), those who could not return to sports (non-coper, NP) (Hartigan et al., 2013) 2 years after ACLR, and healthy controls (HC). To this end, the correlation between the ALFF and ReHo values of different clusters of participants with varied motor abilities and functional test results was examined.

Based on our previous study, we expected to discover more brain function information in Slow4 and Slow5, which maybe more correlated with motor function. Moreover, we expected our findings to identify brain function plasticity characteristics, guide the selection of rehabilitation training exercises, and provide a theoretical basis for later treatment.

## 2 Materials and methods

### 2.1 Study design

A retrospective cohort study was conducted to compare the differences in neural function in patients with different functional abilities 2 years after ACLR. All patients received verbal and written information regarding the study procedures and provided written informed consent for study participation. The Medical Ethics Review Committee of the Second Affiliated Hospital of Zhejiang University School of Medicine approved this study (ethics number: IR2020214) in accordance with the Declaration of the World Medical Association. This study conforms with the Strengthening the Reporting of Observational Studies in Epidemiology guidelines and reports the required information accordingly.

### 2.2 Participants

The inclusion criteria were as follows: age ≥ 18 years but ≤50 years; being right-handed with ACL tears by MRI and arthroscopy and having undergone ACLR by the same surgeon >2 years prior; and having volunteered for an fMRI brain examination. Participants were excluded if they met the following criteria: heart and nervous system diseases, medial collateral ligament injury, meniscus repair, grade III–IV cartilage injury, ankle instability and other sports injuries, metal implants in the body, taking medication, or other diseases.

The additional four inclusion criteria were as follows: (1) number of episodes of knee giving way (≤1); (2) single leg 6-m timed hop index ≥80%; (3) knee outcome survey activities of daily living subscale ≥80%; and (4) global rating scale score, a measure of pre-injury functional level of ≥60%. If a patient failed to meet the established criteria on any of the four tests, the patient was classified as NP, even if adequate scores were achieved on the other three tests (Kapreli et al., 2009). Additionally, we recruited healthy persons as the control group matched with the CP based on sex, age, body mass index (BMI), main power leg, and Tegner scores.

A research team member not involved in data collection conducted all analyses on de-identified data. Overall, 38 patients with ACLR who were hospitalized in the Second Affiliated Hospital of Zhejiang University School of Medicine between January 2019 and December 2021 and met the inclusion criteria were included in this study. Additionally, 15 HCs were recruited. Subsequently, four participants were excluded owing to incomplete cerebellum coverage and excessive head movement, and one person in the HC group was excluded because of an inability to match with the CP. Finally, 17, 17, and 14 participants were allocated to the CP, NP, and HC groups, respectively.

### 2.3 Functional magnetic resonance examination

Data Collection: The same experienced operator performed all scans using a 3.0 T magnetic resonance imaging system (Prisma, Siemens, Germany). Participants were supine on an examination bed, wearing earplugs to reduce external stimulation, with their heads comfortably positioned and fixed. Participants were asked to remain awake and relaxed, with their eyes closed and heads as still as possible. The rs-fMRI sequence scanning parameters were as follows: repetition time = 1,000 ms, echo time = 34 ms, layer thickness = 2.5 mm, layer number = 52, field of view = 230 × 230 mm, matrix = 64 × 64, time point = 360, voxel (2.5 × 2.5 × 2.5 mm), and deflection angle = 90°. The scanning time was 6 min, and during the entire scanning process, the coil was padded with a sponge to limit head movement and ensure the alignment of the rs-fMRI with the structural image.

### 2.4 Functional evaluation

The results of the International Knee Documentation Committee Subjective Knee Evaluation Form (IKDC), Tegner, Knee Injury and Osteoarthritis Outcome Score (KOOS), 6 M timed jump test (Harrison et al., 2017), horizontal jump test (Gustavsson et al., 2006), vertical jump test (Harrison et al., 2017), lateral jump test (Thomeé et al., 2012), and Y-balance test (YBT) (Kim et al., 2023) symmetry were collected. The limb symmetry index was calculated as the affected limb score/healthy limb score × 100 (Thomeé et al., 2012).

### 2.5 Statistical methods

The original Digital Imaging and Communications in Medicine classification was transformed into the Neuroimaging Informatics Technology Initiative format, REST plusv1.25^1^ was utilized for data preprocessing, encompassing time correction, head motion correction, spatial standardization, spatial smoothing, and linear trend regression to pre-characterize the local brain activity of the participants in the resting state.

ALFF was computed based on the Fast Fourier Transform, and the time series of each voxel was converted to the frequency domain without band-pass filtering. The square root was initially calculated at each frequency of the power spectrum, and subsequently, the mean square root was obtained in SFB, Slow4, and Slow5. All individual ALFF maps were computed and normalized into mean ALFF (mALFF). ReHo was calculated for each subject within the different frequency bands. For each voxel, Kendall’s coefficient of concordance was computed between the blood oxygen-level dependent time series for the designated voxel and those of its 26 nearest neighbors, generating a voxel-wise ReHo map for each subject. The individual ReHo map was normalized into subject-level z-score maps by subtracting the mean voxel-wise ReHo obtained for the entire brain (global mean of ReHo) and then dividing by the standard deviation across voxels. Subsequently, a spatial smoothing of 6 mm was executed on the ReHo map.

One-sample t-tests were performed for within-group comparisons in SFB. One-way analysis of variance (ANOVA) were used to compare mALFF and zReHo brain maps among three groups. Two sample t-tests were used to compare mALFF and zReHo brain maps between groups. The Gaussian Random Field theory (GRF) was used for multiple comparison correction (Gong et al., 2018), with voxel *p* < 0.05 and cluster *p* < 0.05 indicating statistical significance. The results were observed using Xjview8. In addition, Pearson’s correlation analysis was performed to assess the relationships between the ALFF and ReHo showing significant differences and clinical variables, with statistical significance set at *p* < 0.05. Moreover, voxel-wise regression analyses of ReHo maps and YBT, Tegner scores were also explored, with voxel *p* < 0.05 and cluster *p* < 0.05 indicating statistical significance.

## 3 Results

Overall, 17 CP, 17 NP, and 14 HC completed the tests. No significant differences were found in age, sex, BMI, and main strength leg among the three groups (*F* > 0.05). No significant differences were observed in the injured limb between the CP and NP groups (*p* > 0.05) (Table 1).

**Table 1:** Demographic data of participants.

	CP (*n* = 17)	NP (*n* = 17)	HC (*n* = 14)	*F/Χ^2^* value	*p* value
Age (years)	30.94 ± 6.16	32.47 ± 6.03	31.13 ± 6.94	0.287	0.752
BMI (kg/m^2^)	22.94 ± 3.56	26.94 ± 9.87	22.73 ± 3.50	1.903	0.162
Sex (female/male)	6/11	6/11	5/9	0.045	0.956
Injured limb (left/right)	12/5	11/6	/	0.356	0.724
Main power leg (left/right)	5/12	7/10	6/8	0.275	0.761

The data are shown as the mean (standard deviation), CP, coper; NP, non-coper; HC, Healthy Controls; BMI, Body Mass Index.

**Figure 1:**
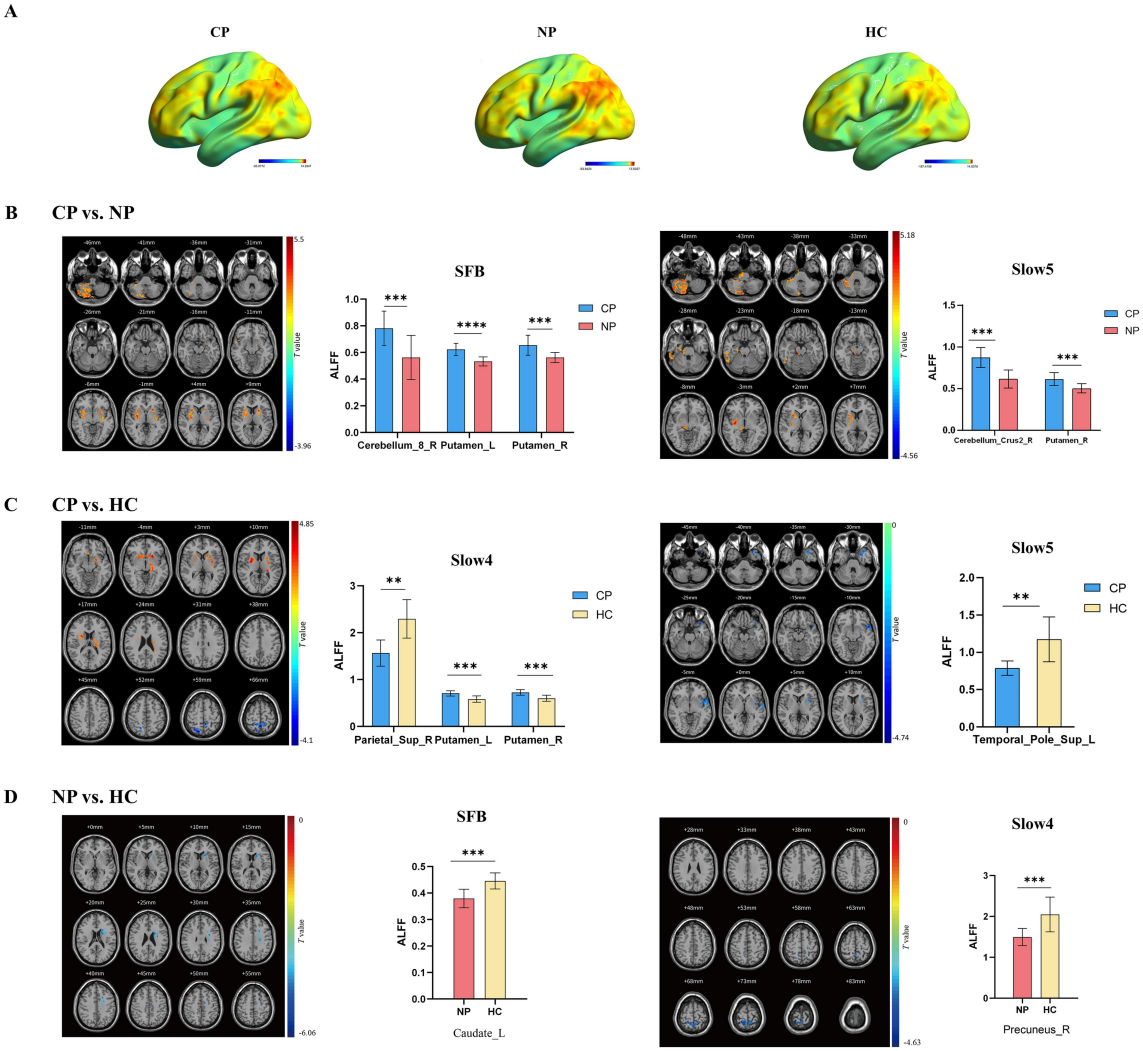
Brain regions exhibiting significant differences in ALFF in different frequency bands between the three groups (voxel *p* < 0.05, cluster *p* < 0.05, GRF correction), along with bar graphs depicting of ALFF values extracted from differential brain regions (*p* < 0.05). (A) ALFF spatial patterns of each group were obtained by uncorrected one-sample *t*-tests. (B) CP vs. NP by two sample *t*-tests (C) CP vs. HC by two sample *t*-tests (D) NP vs. HC by two sample *t*-tests. Warm color indicates the former ALFF values is higher than the latter, and cool color indicates the opposite in brain regions. ALFF, amplitude of low-frequency fluctuations; CP, coper; NP, non-coper; SFB, Standard Frequency Band.

Intrinsic brain activity patterns expressed as normalized group ALFF maps in SFB are shown in Figure 1A (one-sample *t*-test; *p* < 0.05, uncorrected for visual inspection). We found significant differences were among the three groups in the bilateral putamen in SFB, the left putamen and right precuneus in Slow4, and the left lingual gyrus and right caudate in Slow5 (Supplementary Figure S1; Supplementary Table S1). The results of different frequency bands were similar to some extent; and, they were comparable in all aspects. Compared with those of the NP group, the ALLF values of cerebellum_8_R and the bilateral putamen in SFB significantly increased in the CP group, and those of cerebellum_crus2_R and the left putamen in the Slow5 band also significantly increased. The ALLF values of the bilateral putamen in Slow4 were significantly higher, while the parietal lobule in Slow4 and the upper temporal pole in Slow5 were significantly lower in the CP group than in the HC group; The ALLF values of left caudate in SFB and right precuneus in Slow4 were significantly lower in the NP group than in the HC group (voxel *p* < 0.05, cluster *p* < 0.05, GRF correction) (Figure 1; Table 2). Compared with the NP group, the function of the subcortical cerebellum and basal ganglia of the CP group was enhanced, and the motor function of the CP group moved forward from the cortex to the subcortex compared with the HC group, with Slow4 and Slow5 supplementing SFB.

**Table 2:** Brain regions showing significant differences in ALFF in SFB, Slow4, and Slow5.

Frequency	Brain regions (aal)	Cluster size (voxel size)	MNI coordinates			*F/T* value
			X	Y	Z	
CP vs. NP						
SFB	Cerebellum_8_R	299	3	−87	−45	4.5321
	Putamen_L	242	−18	9	18	5.4989
	Putamen_R	317	30	−6	0	4.402
Slow4	NA					
Slow5	Cerebellum_Crus2_R	583	18	−78	−45	5.1783
	Putamen_R	242	30	−15	0	4.5517
CP vs. HC						
SFB	NA					
Slow4	Putamen_R	214	27	−3	9	4.615
	Putamen_L	296	−21	12	−6	4.8514
	Parietal_Sup_R	184	18	−69	57	−4.0754
Slow5	Temporal_Pole_Sup_L	201	−54	12	−9	−4.1481
NP vs. HC						
SFB	Caudate_L	199	−18	9	18	−6.0611
Slow4	Precuneus_R	171	12	−48	75	−4.627
Slow5	NA					

ALFF, amplitude of low-frequency fluctuations; CP, coper; NP, non-coper; HC, Healthy Controls; SFB, Standard Frequency Band; aal, Anatomical Automatic Labeling; MNI, Montreal Neurological Institute.

**Figure 2:**
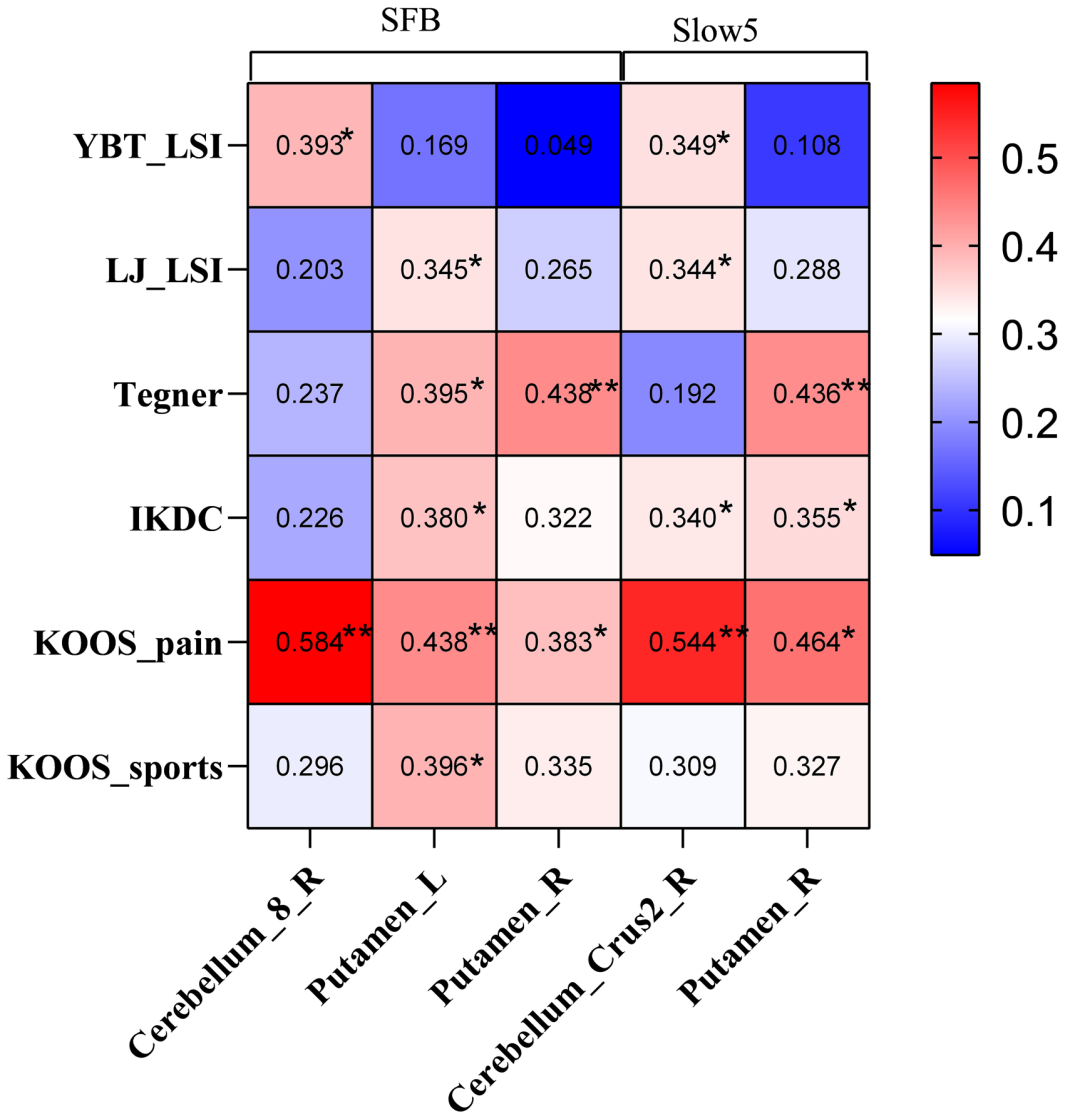
Heat map illustrating clinical correlations between ALFF values with significant differences and functional evaluations in the CP and NP groups. YBT, Y-balance test; LSI: Limb symmetry index; LJ, Lateral jump test; IKDC: International Knee Documentation Committee Subjective Knee Evaluation Form; KOOS, Knee Injury and Osteoarthritis Outcome Score; SFB, Standard Frequency Band; ALFF, amplitude of low-frequency fluctuations.

Regarding functional test results, we selected the ALFF values from brain areas showing differences between the CP and NP groups. Subsequently, we conducted Pearson correlation analysis with the results of YBT-LSI, LJ-LSI, Tegner, IKDC, KOOS-pain, and KOOS-sports, among others, used to evaluate the ability to return to sports. The results were presented using a heat map. The correlation analysis between the ALFF values obtained from differential clusters and the functional evaluation results indicated that YBT results were correlated with ALFF in the cerebellum; Tegner was moderately correlated with ALFF in the putamen; and LJ, IKDC, and KOOS were correlated with ALFF in the cerebellum and putamen, especially with KOOS-pain (Figure 2).

**Figure 3:**
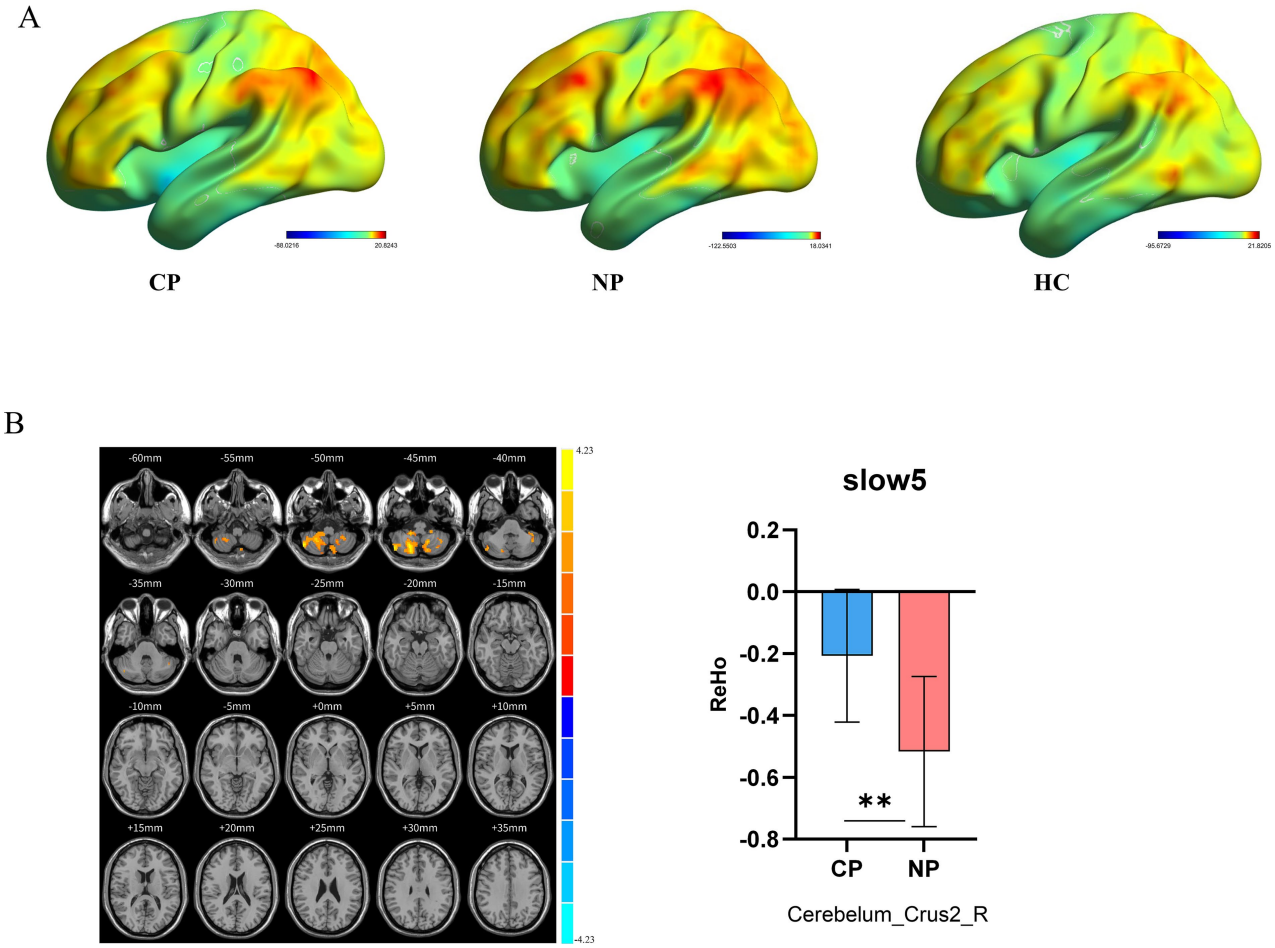
Brain regions revealing significant differences in ReHo in different frequency bands between the three groups. (A) ReHo spatial patterns of each group were obtained by uncorrected one-sample *t*-tests. (B) CP vs. NP by two sample t-tests in Slow5 (voxel *p* < 0.05, cluster *p* < 0.05, GRF correction) and bar graph of ReHo values extracted from differential brain regions (*p* < 0.05). Warm color indicates higher ReHo values in CP than NP, and cool color indicates the opposite ReHo, Regional Homogeneity; CP, coper; NP, non-coper; HC, Healthy Controls; GRF, Gaussian Random Field theory.

Intrinsic brain activity patterns expressed as normalized group ReHo maps in SFB are shown in Figure 3A (one-sample t-test; *p* < 0.05, uncorrected for visual inspection). The ReHo values of left putamen in SFB and right cerebellum_8 regions, left putamen, right caudate nucleus, and right middle cingulate in Slow5 of all groups were significant (Supplementary Figure S2; Supplementary Table S2). The ReHo value of the CP group in the right cerebellum_crus2 was higher than that in the NP group (voxel *p* < 0.05, cluster *p* < 0.05, GRF correction) (Figure 3B; Table 3). CP vs. HC and NP vs. HC had no significant difference. The results showed that the CP group had more synchronization of neuronal activity in the cerebellum. Reho was consistent with ALFF, especially in Slow5.

**Table 3:** Brain regions showing significant differences in ReHo in SFB, Slow4, and Slow5.

Frequency	Brain regions (aal)	Cluster size (voxel size)	MNI coordinates			*T* value
			X	Y	Z	
Slow5	Cerebelum_Crus2_R	575	51	−66	−48	4.2348

ReHo, Regional Homogeneity; CP, coper; NP, non-coper; HC, Healthy Controls; SFB, Standard Frequency Band; aal, Anatomical Automatic Labeling; MNI, Montreal Neurological Institute; NA, not available.

**Figure 4:**
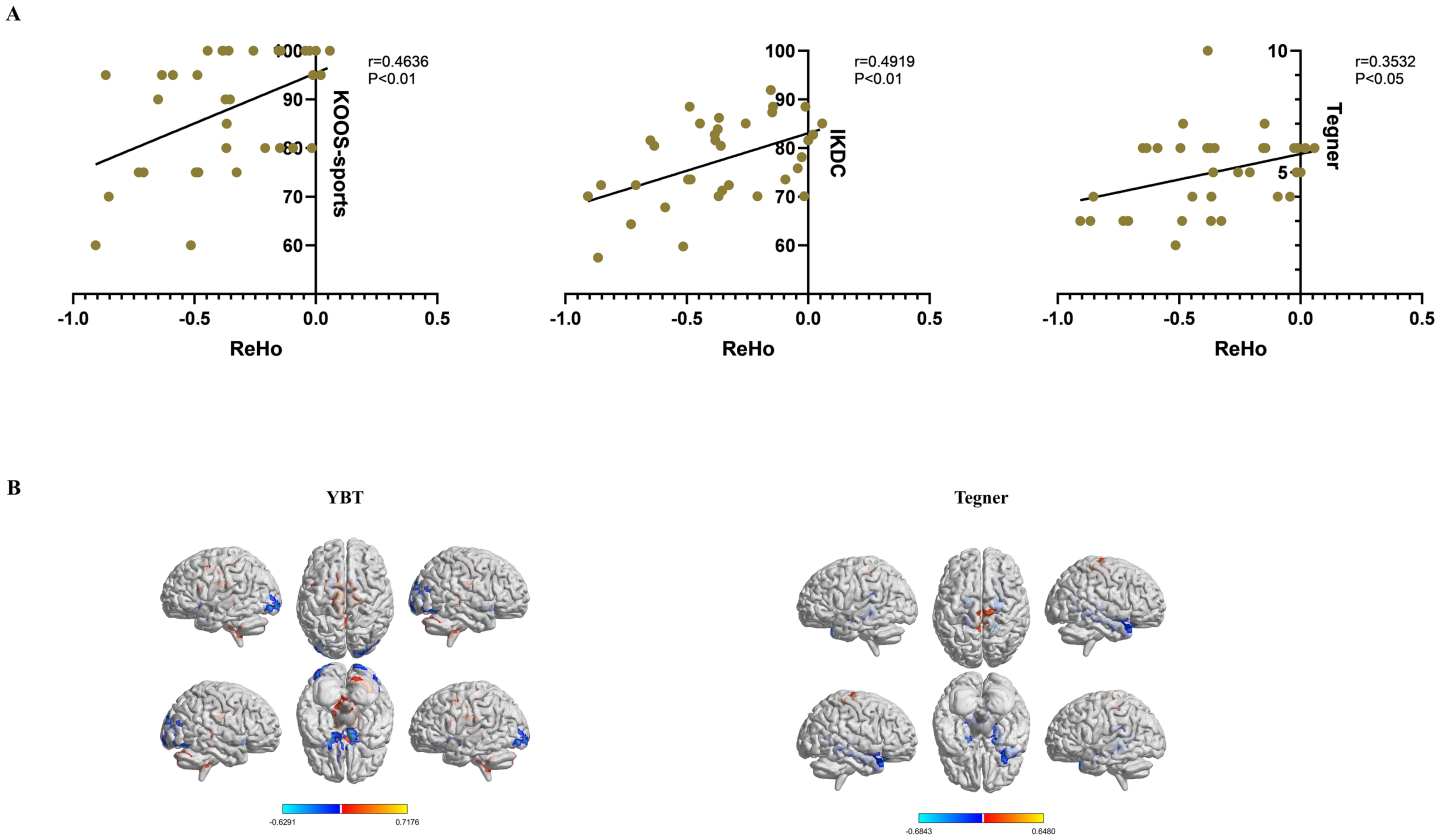
Clinical correlations between ReHo values with significant differences and functional evaluations in the CP and NP groups. (A) Pearson’s correlation analysis between ReHo values of the right cerebelum_crus2 and functional evaluations (*p* < 0.05). (B) Whole-brain voxel-wise regression analysis of ReHo in Slow5 with YBT-LSI, Tegner. Warm color indicates a positive correlation, and cool color indicates the opposite (voxel *p* < 0.05, cluster *p* < 0.05, GRF correction). ReHo, Regional Homogeneity; CP, coper; NP, non-coper; HC, Healthy Controls; GRF, Gaussian Random Field theory. YBT, Y-balance test; LSI, Limb symmetry index.

Regarding ReHo values and functional assessments, KOOS-sports, IKDC and Tegner scores exhibited significant correlations with ReHo values of right cerebellum_crus2 (*p* < 0.05) (Figure 4A). Additionally, we performed a regression analysis of whole brain ReHo values in Slow 5 with YBT-LSI and Tegner to explore alternative correlations between brain function and behavioral indices; the results were interesting: YBT-LSI was positively correlated with ReHo in vermis_4_5, right cerebellum_crus2, left caudate, and left middle cingulate, and negatively correlated with ReHo in inferior occipital and left rectus. Conversely, Tegner was positively correlated with ReHo in the right supplementary motor area (SMA) and negatively correlated with ReHo in the bilateral para-hippocampal gyrus in Slow5 (Figure 4B; Table 4). Although both analyses are correlational in nature: one at the voxel level while the other represents average brain regain values, they exhibit distinct differences. Overall, YBT is associated primarily with cerebellar function whereas Tegner relates more closely to putamen activity as well as SMA and cerebellar functions.

**Table 4:** Correlation of ReHo with pat group and YBT-LSI, Tegner in Slow5, GRF correction, voxel *p* < 0. 05, and cluster *p* < 0.05.

Contrast	Brain regions	Cluster size	MNI coordinates			*r*-value
			X	Y	Z	
YBT-LSI						
Positive correlation	Vermis_4_5	293	0	−54	−27	0.71756
	Cerebelum_Crus2_R	157	21	−81	−39	0.61896
	Caudate_L	183	−12	−6	27	0.65065
	Cingulum_Mid_L	139	−3	−15	45	0.64335
Negative correlation	Occipital_Inf_R	236	42	−72	−15	−0.57059
	Rectus_L	172	0	15	−15	−0.62912
	Occipital_Inf_L	159	−39	−75	−3	−0.56315
Tegner						
Positive correlation	Supp_Motor_Area_R	169	12	−18	63	0.64802
Negative correlation	ParaHippocampal_R	379	21	−42	−9	−0.63747
	ParaHippocampal_L	211	−24	−33	−9	−0.68433

YBT, Y-balance test; LSI: Limb symmetry index; MNI, Montreal Neurological Institute.

Furthermore, we completed several samples of time series plots, including BOLD signal time series that facilitate the visualization of raw data prior to any processing steps; ReHo Time Series Plots that demonstrate how ReHo fluctuates over time across different subjects; and Mean Time Series that aid in visualizing average brain activity patterns and their variations among three groups (Supplementary Figure S3).

## 4 Discussion

To the best of our knowledge, this is the first study to systematically evaluate ALFF and ReHo values in SFB, Slow4, and Slow5 frequency bands separately among people with different motor abilities after ACLR surgery. Our findings suggest differential brain function performance in people with different motor abilities and that subcortical function migration in the CP group is conducive to their return to sports. The ALFF value is consistent with the ReHo value. YBT is associated primarily with cerebellar function whereas Tegner relates more closely to putamen activity as well as SMA and cerebellar functions.

Previous studies on return to sports have tended to focus on task-fMRI. Kapreli et al. (2009) first used task-fMRI to demonstrate that, compared to controls, patients with ACL deficiency had diminished activity in several sensorimotor cortical areas and increased activity in the following three areas: pre-SMA, posterior secondary somatosensory area, and posterior inferior temporal gyrus. Grooms et al. (2017) compared patients with ACLR who could return to sports 6 months to 5 years postoperatively with healthy matched controls. They noted that participants who had undergone ACLR had increased activity in the contralateral motor cortex, lingual gyrus, and ipsilateral secondary somatosensory area and diminished activation in the ipsilateral motor cortex and cerebellum compared to healthy matched controls. The findings may indicate a shift toward a visual-motor strategy as opposed to a sensory-motor strategy to engage in knee movement. Criss et al. (2023) conducted task-fMRI studies on patients who returned to exercise 2–6 years after ACLR surgery and demonstrated that the function of the ipsilateral secondary somatosensory cortex and the ipsilateral SMA increased. In our study, rs-fMRI was used to observe patients for >2 years postoperatively, and the subcortical function, such as that of the cerebellum and putamen, was increased in those who returned to sports, while the motor cortex function was decreased. Although task-fMRI is a study of brain areas immediately activated by the task paradigm at that time, some potential motor functions and planning abilities may be reflected in rs-fMRI, which might explain why the results of this study are different from those of previous studies.

There are relatively few studies on rs-fMRI regarding ACLR. Wang et al. (2019) revealed that the ALFF in the brain was significantly higher than that in the control group in the bilateral central cingulate gyrus and involved the SMAs. The fALFF in activation cluster 1 was significantly higher in the right central gyrus, right lower lobule, and right upper margin in patients than that in the normal control group; the fALFF in activation cluster 2 in the right central cingulate gyrus, involving the right supplementary movement zone was significantly higher in patients than that in the normal control group. This study was conducted within 3 months postoperatively and reflected changes in the sensorimotor cortex of the brain, while the patients did not return to sports; further observations of brain function remodeling after returning to sports are needed. Therefore, our study was based on patients who had undergone the surgery 2 years prior, which can better reflect the brain regions more relevant to return to sports. For the first time, we used the ReHo calculation method to explore the consistency of brain functional regions after ACLR surgery. The study results indicated a significant difference in the cerebellum_rus2 region when CP was compared with NP, which was consistent with the results of the ALFF value in Slow5, suggesting that neurons excited themselves while diffusing excitability outward. This makes it possible to complete some complex sports.

Located in the dorsal fossa of the brain stem, the cerebellum has multiple connections with the brain, brain stem, and spinal cord. It is crucial in motor control, motor coordination, and learning of fine motor skills (Roostaei et al., 2014). The classic basal ganglia model describes how information returns to the cerebral cortex through two neural pathways in the basal ganglia to achieve normal regulation of movement (Sheng et al., 2019). However, the basal ganglia are involved in more complex goal-directed behaviors (Haber, 2016), including emotion, motivation, and the cognitive components that express specific movements, which are highly correlated with higher motor functions. Redundancy in the system enables adaptation and compensation when sensory patterns are impaired. These behavioral changes may be learned through reward- and error-based learning processes implemented via the basal ganglia and cerebellum pathways, respectively (Roostaei et al., 2014).

Previous studies on the correlation between ALFF values and functional test results or functional scores of differential brain masses are scarce. Lepley et al. (2019) revealed that KOOS-pain, and symptoms were significantly correlated with increased activation of the frontal lobe of the brain using task-fMRI. Criss et al. (2023) found that patient-reported knee function was positively and moderately correlated with the ipsilateral secondary somatosensory cortex and the ipsilateral SMA. Our study also demonstrated that KOOS-pain was moderately correlated with increased activation of subcortical brain function. YBT results were correlated with cerebellum ALFF values, and Tegner was moderately correlated with putamen ALFF values. Simultaneously, when we calculated the ReHo value in the Slow5 band and performed regression analysis with YBT and Tegner, we also found that vermis_4_5, cerebellum_crus2, caudate nucleus, and middle cingulate gyrus were positively correlated with YBT function, while the SMA was positively correlated with Tegner. These functional evaluation results may indirectly reflect the function of brain areas and can be recommended for clinical evaluation of return to sports.

Buzsaki et al. proposed the sub-band theory to reduce the omission of information from brain regions (Zuo et al., 2010; Buzsáki and Draguhn, 2004). Previous studies focused on Slow4 and Slow5, and the results revealed that brain ALFF in different frequency bands had band-specific neural functional changes (Wu et al., 2023; Yang et al., 2022). The same characteristics were also demonstrated in this study. First, in comparing the three groups, significant differences were found in the bilateral putamen in the SFB. In contrast, differences in the right precuneus in Slow4 and left lingual gyrus and right caudate in Slow5 were explored. In the comparison between the CP and NP groups and between the CP and HC groups, we also obtained functions different from those in the SFB and obtained reduced information on the sensorimotor cortex in the CP group. Therefore, the study of the sub-frequency band reflects certain band specificity and warrants further investigation.

This study had some limitations. First, only part of the calculation method of rs-fMRI was used in this study, and other calculation methods can be employed to explore the imaging features that can characterize the return to sports of ACLR. Second, the sample size of this study was small, and the study was conducted retrospectively. Although patients who had undergone rehabilitation were selected as far as possible, whether they had received normative rehabilitation treatment cannot be guaranteed. Third, although our study layer thickness = 2.5 and time point = 360, the overall test time was short, which may affect the reliability of the study.

Subsequent studies will continue to confirm these findings on a larger sample, with longer scan duration and better spatiotemporal resolution. Additionally, to increase prospective observation or therapeutic studies, further studies will explore rehabilitation treatment methods conducive to return to sports.

In conclusion, our study shows that there are significant differences in brain function remodeling in patients with ACLR with different motor abilities 2 years post-operation, which is correlated with clinical function test indicators. ALFF results suggested that brain function in the CP group shifted to the subcortical basal ganglia and cerebellum, while ReHo results suggested that cerebellum synchronization of neuronal activity in the CP group was increased. These changes in brain function may be the reason for postoperative patients to return to exercise. Postoperative rehabilitation can strengthen the training of subcortical function and improve the return-to-sport rate of patients with ACLR. Slow4 and Slow5 complement the SFB and warrant further study.

## Data Availability

The raw data supporting the conclusions of this article will be made available by the authors, without undue reservation.

## Footnotes

^1^ http://www.restfmri.net/forum/restplus

## References

[ref1] An Y. W. DiTrani Lobacz A. Lehmann T. Baumeister J. Rose W. C. Higginson J. S.. (2018). Neuroplastic changes in ACLR patients from neuromechanical decoupling. Scand. J. Med. Sci. Sports 29, 251–258. doi: 10.1111/sms.13322, PMID: 30326547

[ref2] Ardern C. L. Taylor N. F. Feller J. A. Webster K. E. (2014). Fifty-five per cent return to competitive sport following anterior cruciate ligament reconstruction surgery: an updated systematic review and meta-analysis including aspects of physical functioning and contextual factors. Br. J. Sports Med. 48, 1543–1552. doi: 10.1136/bjsports-2013-093398 25157180

[ref3] Baumeister J. Reinecke K. Schubert M. Weiss M. (2011). Altered electrocortical brain activity after ACL reconstruction during force control. J. Orthop. Res. 29, 1383–1389. doi: 10.1002/jor.21380, PMID: 21437965

[ref4] Baumeister J. Reinecke K. Weiss M. (2008). Changed cortical activity after anterior cruciate ligament reconstruction in a joint position paradigm: An eeg study. Scand. J. Med. Sci. Sports 18, 473–484. doi: 10.1111/j.1600-0838.2007.00702.x, PMID: 18067525

[ref5] Buzsáki G. Draguhn A. (2004). Neuronal oscillations in cortical networks. Science 304, 1926–1929. doi: 10.1126/science.1099745 15218136

[ref6] Criss C. R. Lepley A. S. Onate J. A. Simon J. E. France C. R. Clark B. C.. (2023). Neural correlates of self-reported knee function in individuals after anterior cruciate ligament reconstruction. Sports Health 15, 52–60. doi: 10.1177/19417381221079339, PMID: 35321615 PMC9808834

[ref7] Fox M. D. Raichle M. E. (2007). Spontaneous fluctuations in brain activity observed with functional magnetic resonance imaging. Nat. Rev. Neurosci. 8, 700–711. doi: 10.1038/nrn2201 17704812

[ref8] Gong W. Wan L. Lu W. Ma L. Cheng F. Cheng W.. (2018). Statistical testing and power analysis for brain-wide association study. Med. Image Anal. 47, 15–30. doi: 10.1016/j.media.2018.03.014, PMID: 29656107

[ref9] Grooms D. R. Page S. J. Nichols-Larsen D. S. Chaudhari A. M. White S. E. Onate J. A. (2017). Neuroplasticity associated with anterior cruciate ligament reconstruction. J. Orthop. Sports Phys. Ther. 47, 180–189. doi: 10.2519/jospt.2017.7003, PMID: 27817301

[ref10] Gustavsson A. Neeter C. Thomeé P. Silbernagel K. G. Augustsson J. Thomeé R.. (2006). A test battery for evaluating hop performance in patients with an ACL injury and patients who have undergone ACL reconstruction. Knee Surg. Sports Traumatol. Arthrosc. 14, 778–788. doi: 10.1007/s00167-006-0045-6, PMID: 16525796

[ref11] Haber S. N. (2016). Corticostriatal circuitry. Dialogues Clin. Neurosci. 18, 7–21. doi: 10.31887/DCNS.2016.18.1/shaber, PMID: 27069376 PMC4826773

[ref12] Harrison J. J. Yorgey M. K. Csiernik A. J. Vogler J. H. Games K. E. (2017). Clinician-friendly physical performance tests for the knee. J. Athl. Train. 52, 1068–1069. doi: 10.4085/1062-6050-52.11.19, PMID: 29116831 PMC5737044

[ref13] Hartigan E. H. Lynch A. D. Logerstedt D. S. Chmielewski T. L. Snyder-Mackler L. (2013). Kinesiophobia after anterior cruciate ligament rupture and reconstruction: noncopers versus potential copers. J. Orthop. Sports Phys. Ther. 43, 821–832. doi: 10.2519/jospt.2013.4514, PMID: 24175594 PMC4915102

[ref14] Kapreli E. Athanasopoulos S. Gliatis J. Papathanasiou M. Peeters R. Strimpakos N.. (2009). Anterior cruciate ligament deficiency causes brain plasticity: A functional MRI study. Am. J. Sports Med. 37, 2419–2426. doi: 10.1177/0363546509343201 19940314

[ref15] Kim J. G. Lee D. W. Bae K. C. Choi B. C. Yang S. J. Cho S. I.. (2023). Correlation of y balance with clinical scores and functional tests after anterior cruciate ligament reconstruction in young and middle-aged patients. Clin. Orthop. Surg. 15, 50–58. doi: 10.4055/cios21131, PMID: 36778986 PMC9880508

[ref16] Lepley A. S. Grooms D. R. Burland J. P. Davi S. M. Kinsella-Shaw J. M. Lepley L. K. (2019). Quadriceps muscle function following anterior cruciate ligament reconstruction: systemic differences in neural and morphological characteristics. Exp. Brain Res. 237, 1267–1278. doi: 10.1007/s00221-019-05499-x, PMID: 30852644

[ref17] Luc-Harkey B. A. Harkey M. S. Pamukoff D. N. Kim R. H. Royal T. K. Blackburn J. T.. (2017). Greater intracortical inhibition associates with lower quadriceps voluntary activation in individuals with ACL reconstruction. Exp. Brain Res. 235, 1129–1137. doi: 10.1007/s00221-017-4877-8 28144695

[ref18] Paterno M. V. Rauh M. J. Schmitt L. C. Ford K. R. Hewett T. E. (2014). Incidence of second ACL injuries 2 years after primary ACL reconstruction and return to sport. Am. J. Sports Med. 42, 1567–1573. doi: 10.1177/0363546514530088, PMID: 24753238 PMC4205204

[ref19] Pietrosimone B. G. Lepley A. S. Ericksen H. M. Clements A. Sohn D. H. Gribble P. A. (2015). Neural excitability alterations after anterior cruciate ligament reconstruction. J. Athl. Train. 50, 665–674. doi: 10.4085/1062-6050-50.1.11, PMID: 25844855 PMC4527451

[ref20] Roostaei T. Nazeri A. Sahraian M. A. Minagar A. (2014). The human cerebellum: a review of physiologic neuroanatomy. Neurol. Clin. 32, 859–869. doi: 10.1016/j.ncl.2014.07.013 25439284

[ref21] Sheng M. J. Lu D. Shen Z. M. Poo M. M. (2019). Emergence of stable striatal d1r and d2r neuronal ensembles with distinct firing sequence during motor learning. Proc. Natl. Acad. Sci. USA 116, 11038–11047. doi: 10.1073/pnas.1901712116, PMID: 31072930 PMC6561210

[ref22] Thomeé R. Neeter C. Gustavsson A. Thomeé P. Augustsson J. Eriksson B.. (2012). Variability in leg muscle power and hop performance after anterior cruciate ligament reconstruction. Knee Surg. Sports Traumatol. Arthrosc. 20, 1143–1151. doi: 10.1007/s00167-012-1912-y 22314862

[ref23] Wang J. Q. Liu H. Wang X. B. Zhang Y. Q. Wang S. Q. Shi Y. Q.. (2019). A preliminary study on resting-state functional magnetic resonance imaging of brain after anterior cruciate ligament preservation reconstruction with autologous tendon. Zhonghua Yi Xue Za Zhi 99, 1479–1483. doi: 10.3760/cma.j.issn.0376-2491.2019.19.009, PMID: 31137138

[ref24] Wu X. Wang L. Jiang H. Fu Y. Wang T. Ma Z.. (2023). Frequency-dependent and time-variant alterations of neural activity in post-stroke depression: a resting-state fmri study. Neuroimage Clin. 38:103445. doi: 10.1016/j.nicl.2023.103445, PMID: 37269698 PMC10244813

[ref25] Yang H. Zhang H. Meng C. Wohlschlager A. Brandl F. Di X.. (2022). Frequency-specific coactivation patterns in resting-state and their alterations in schizophrenia: An fmri study. Hum. Brain Mapp. 43, 3792–3808. doi: 10.1002/hbm.25884, PMID: 35475569 PMC9294298

[ref26] Zang Y. F. He Y. Zhu C. Z. Cao Q. J. Sui M. Q. Liang M.. (2007). Altered baseline brain activity in children with adhd revealed by resting-state functional mri. Brain and Development 29, 83–91. doi: 10.1016/j.braindev.2006.07.002, PMID: 16919409

[ref27] Zang Y. Jiang T. Lu Y. He Y. Tian L. (2004). Regional homogeneity approach to fmri data analysis. NeuroImage 22, 394–400. doi: 10.1016/j.neuroimage.2003.12.030 15110032

[ref28] Zou Q. H. Zhu C. Z. Yang Y. Zuo X. N. Long X. Y. Cao Q. J.. (2008). An improved approach to detection of amplitude of low-frequency fluctuation (alff) for resting-state fmri: fractional alff. J. Neurosci. Methods 172, 137–141. doi: 10.1016/j.jneumeth.2008.04.012, PMID: 18501969 PMC3902859

[ref29] Zuo X. N. Di Martino A. Kelly C. Shehzad Z. E. Gee D. G. Klein D. F.. (2010). The oscillating brain: complex and reliable. NeuroImage 49, 1432–1445. doi: 10.1016/j.neuroimage.2009.09.037, PMID: 19782143 PMC2856476

