# Supplementary material for: Neural functions vary by return-to-sport status in participants with anterior cruciate ligament reconstruction: a retrospective cohort study using sub-bands of resting-state functional magnetic resonance
Source: Front Hum Neurosci. 2024 Nov 1;18:1457823. doi: 10.3389/fnhum.2024.1457823 (PMC11564169; doi:10.3389/fnhum.2024.1457823)
Supplement: Supplementary file 4 [file Presentation_1.PPTX]

## Slide 1
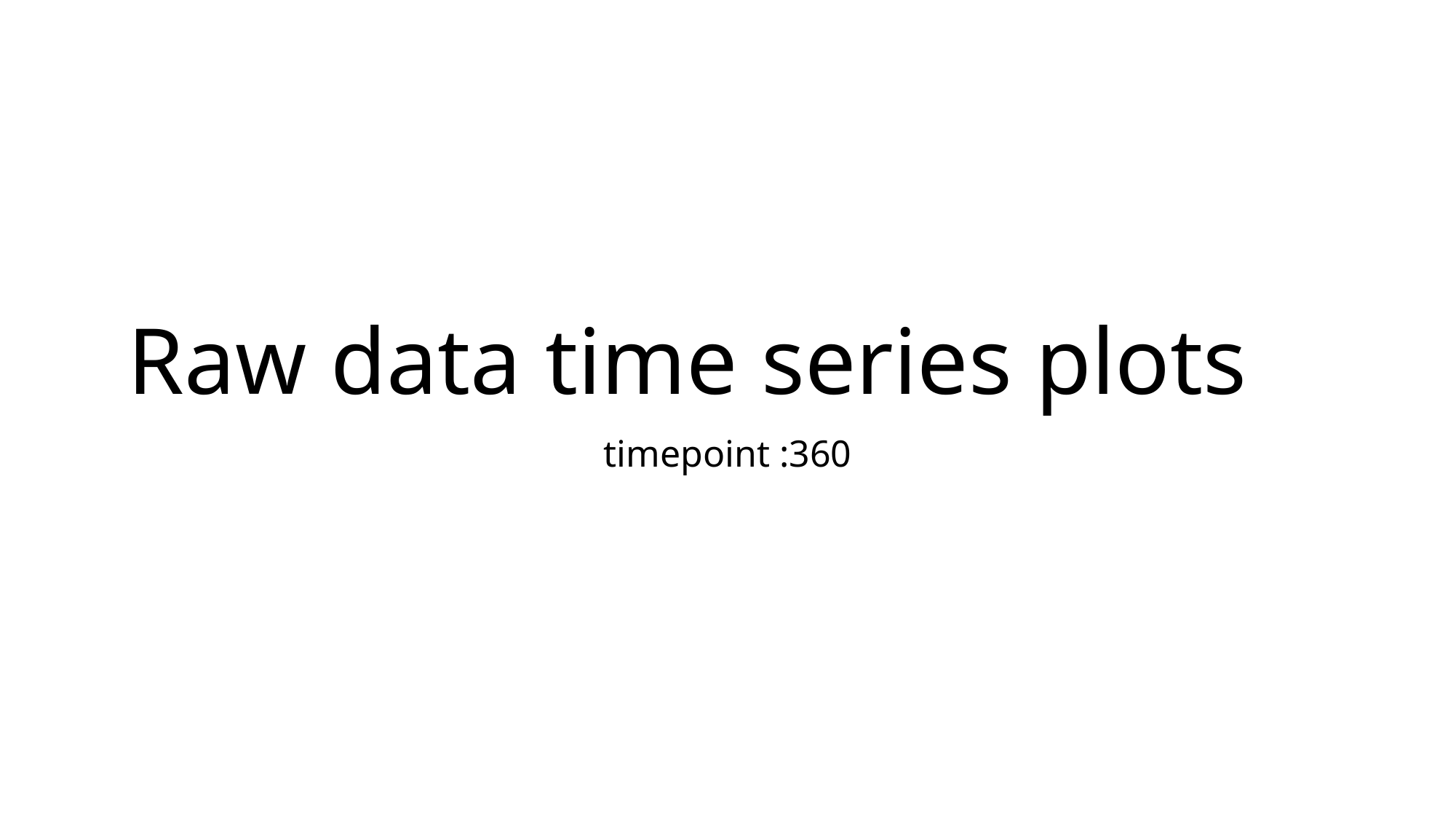

# Raw data time series plots
timepoint :360

## Slide 2
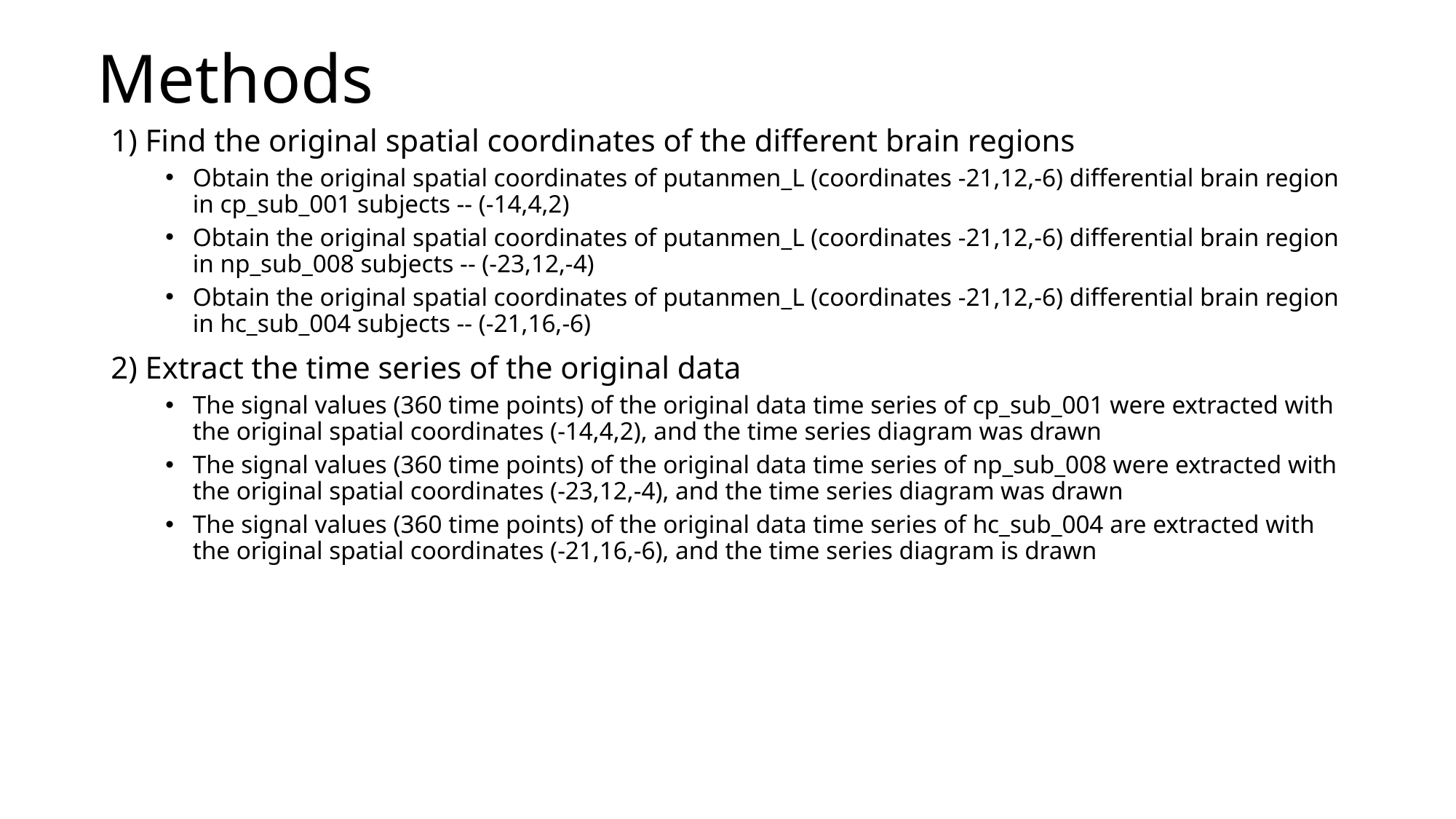

# Methods
1) Find the original spatial coordinates of the different brain regions
Obtain the original spatial coordinates of putanmen_L (coordinates -21,12,-6) differential brain region in cp_sub_001 subjects -- (-14,4,2)
Obtain the original spatial coordinates of putanmen_L (coordinates -21,12,-6) differential brain region in np_sub_008 subjects -- (-23,12,-4)
Obtain the original spatial coordinates of putanmen_L (coordinates -21,12,-6) differential brain region in hc_sub_004 subjects -- (-21,16,-6)
2) Extract the time series of the original data
The signal values (360 time points) of the original data time series of cp_sub_001 were extracted with the original spatial coordinates (-14,4,2), and the time series diagram was drawn
The signal values (360 time points) of the original data time series of np_sub_008 were extracted with the original spatial coordinates (-23,12,-4), and the time series diagram was drawn
The signal values (360 time points) of the original data time series of hc_sub_004 are extracted with the original spatial coordinates (-21,16,-6), and the time series diagram is drawn

## Slide 3
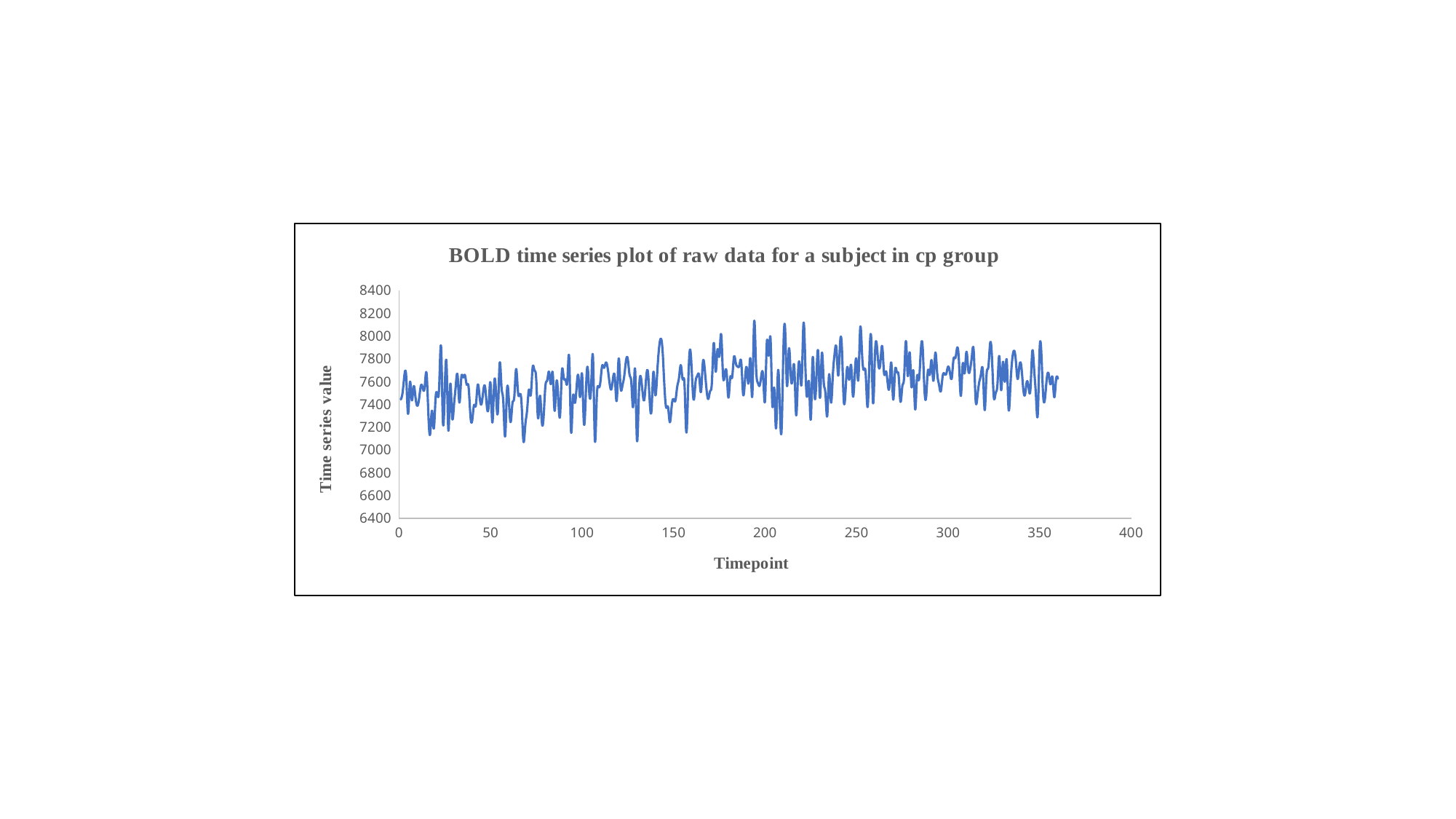

### Chart: BOLD time series plot of raw data for a subject in cp group
| Category | cp_sub_001 |
|---|---|

## Slide 4
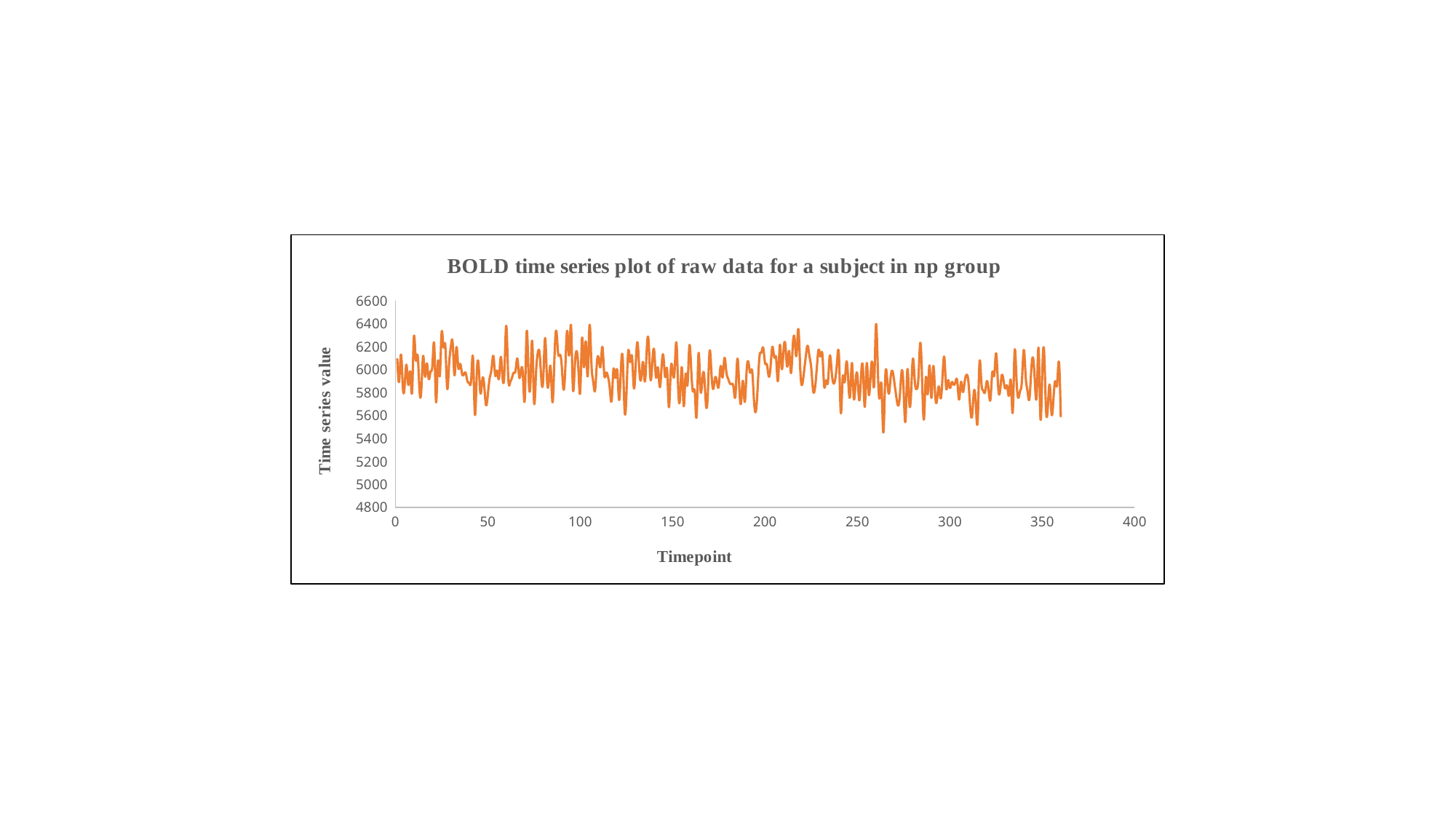

### Chart: BOLD time series plot of raw data for a subject in np group
| Category | np_sub_008 |
|---|---|

## Slide 5
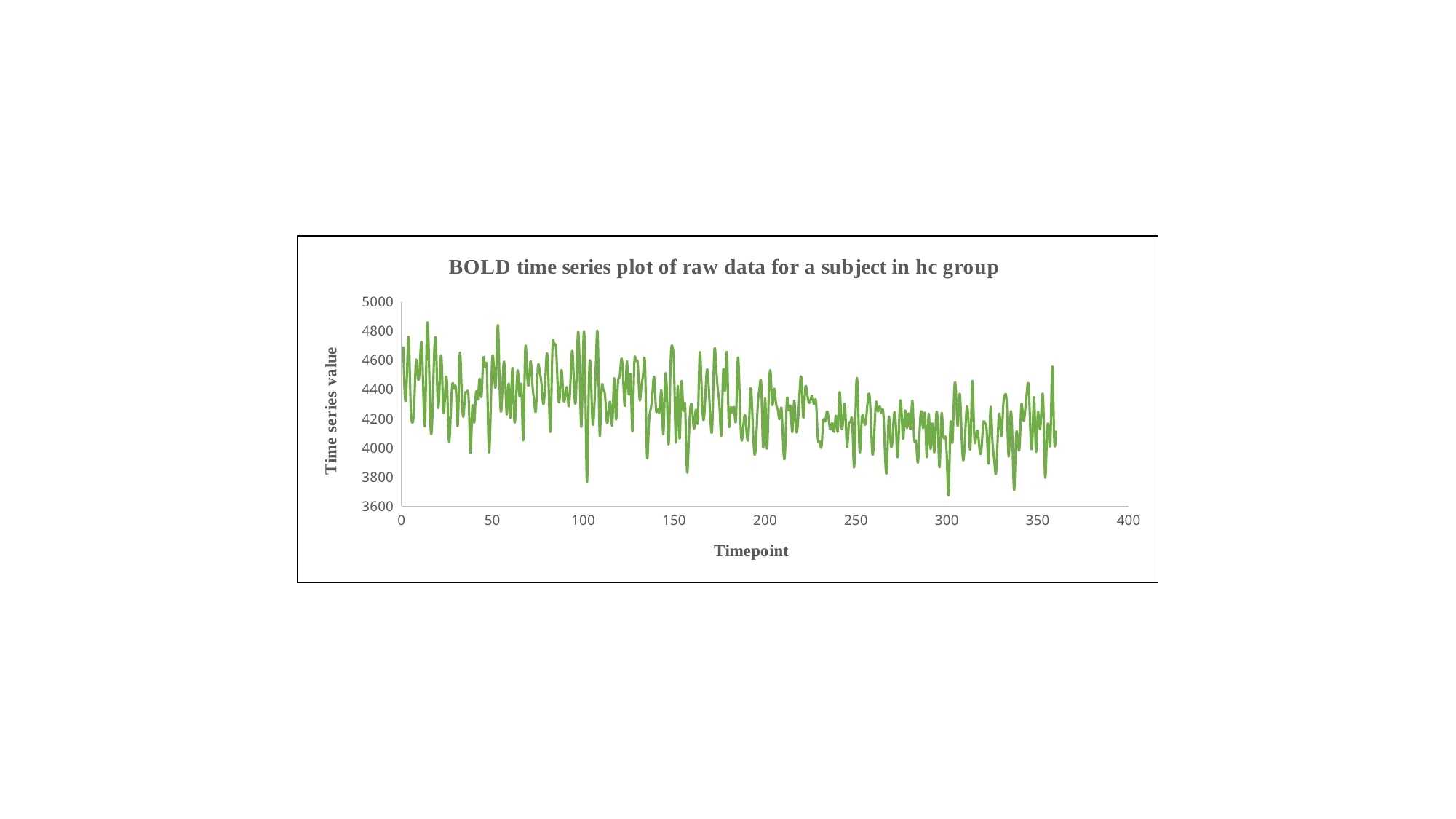

### Chart: BOLD time series plot of raw data for a subject in hc group
| Category | hc_sub_004 |
|---|---|
