# Supplementary material for: Neural functions vary by return-to-sport status in participants with anterior cruciate ligament reconstruction: a retrospective cohort study using sub-bands of resting-state functional magnetic resonance
Source: Front Hum Neurosci. 2024 Nov 1;18:1457823. doi: 10.3389/fnhum.2024.1457823 (PMC11564169; doi:10.3389/fnhum.2024.1457823)
Supplement: Supplementary file 5 [file Presentation_2.PPTX]

## Slide 1
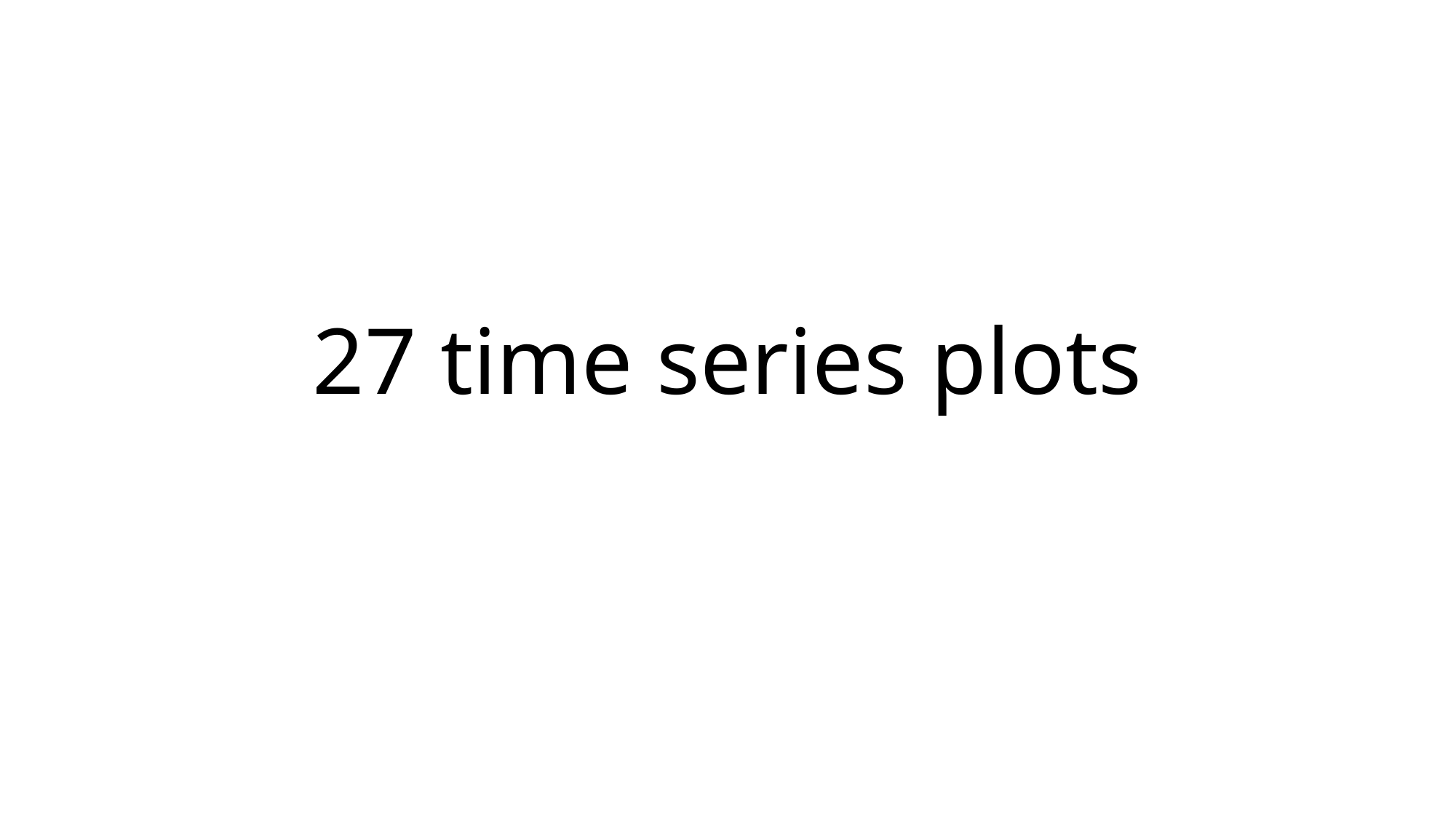

# 27 time series plots

## Slide 2
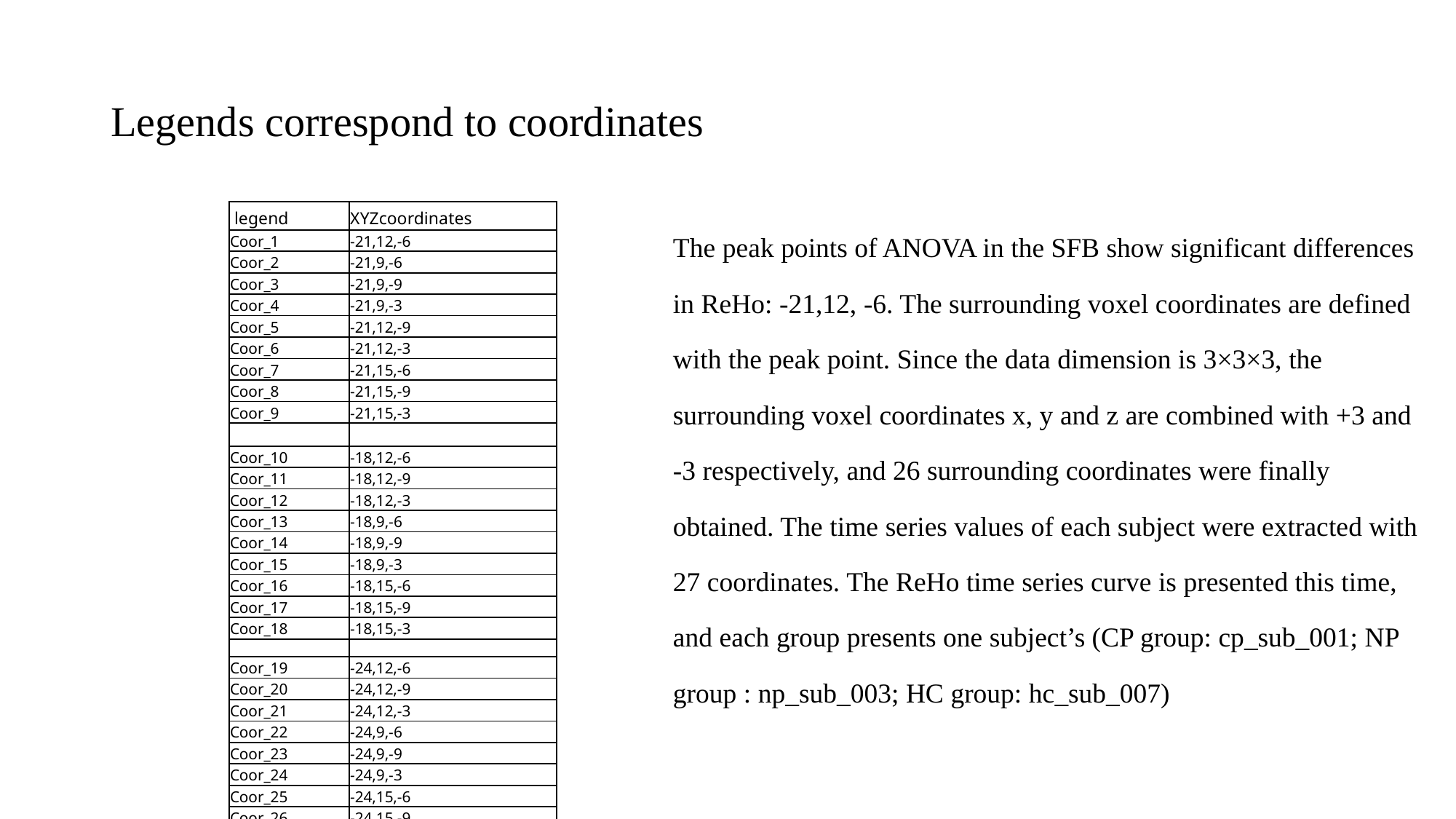

# Legends correspond to coordinates
| legend | XYZcoordinates |
| --- | --- |
| Coor\_1 | -21,12,-6 |
| Coor\_2 | -21,9,-6 |
| Coor\_3 | -21,9,-9 |
| Coor\_4 | -21,9,-3 |
| Coor\_5 | -21,12,-9 |
| Coor\_6 | -21,12,-3 |
| Coor\_7 | -21,15,-6 |
| Coor\_8 | -21,15,-9 |
| Coor\_9 | -21,15,-3 |
| | |
| Coor\_10 | -18,12,-6 |
| Coor\_11 | -18,12,-9 |
| Coor\_12 | -18,12,-3 |
| Coor\_13 | -18,9,-6 |
| Coor\_14 | -18,9,-9 |
| Coor\_15 | -18,9,-3 |
| Coor\_16 | -18,15,-6 |
| Coor\_17 | -18,15,-9 |
| Coor\_18 | -18,15,-3 |
| | |
| Coor\_19 | -24,12,-6 |
| Coor\_20 | -24,12,-9 |
| Coor\_21 | -24,12,-3 |
| Coor\_22 | -24,9,-6 |
| Coor\_23 | -24,9,-9 |
| Coor\_24 | -24,9,-3 |
| Coor\_25 | -24,15,-6 |
| Coor\_26 | -24,15,-9 |
| Coor\_27 | -24,15,-3 |
The peak points of ANOVA in the SFB show significant differences in ReHo: -21,12, -6. The surrounding voxel coordinates are defined with the peak point. Since the data dimension is 3×3×3, the surrounding voxel coordinates x, y and z are combined with +3 and -3 respectively, and 26 surrounding coordinates were finally obtained. The time series values of each subject were extracted with 27 coordinates. The ReHo time series curve is presented this time, and each group presents one subject’s (CP group: cp_sub_001; NP group : np_sub_003; HC group: hc_sub_007)

## Slide 3
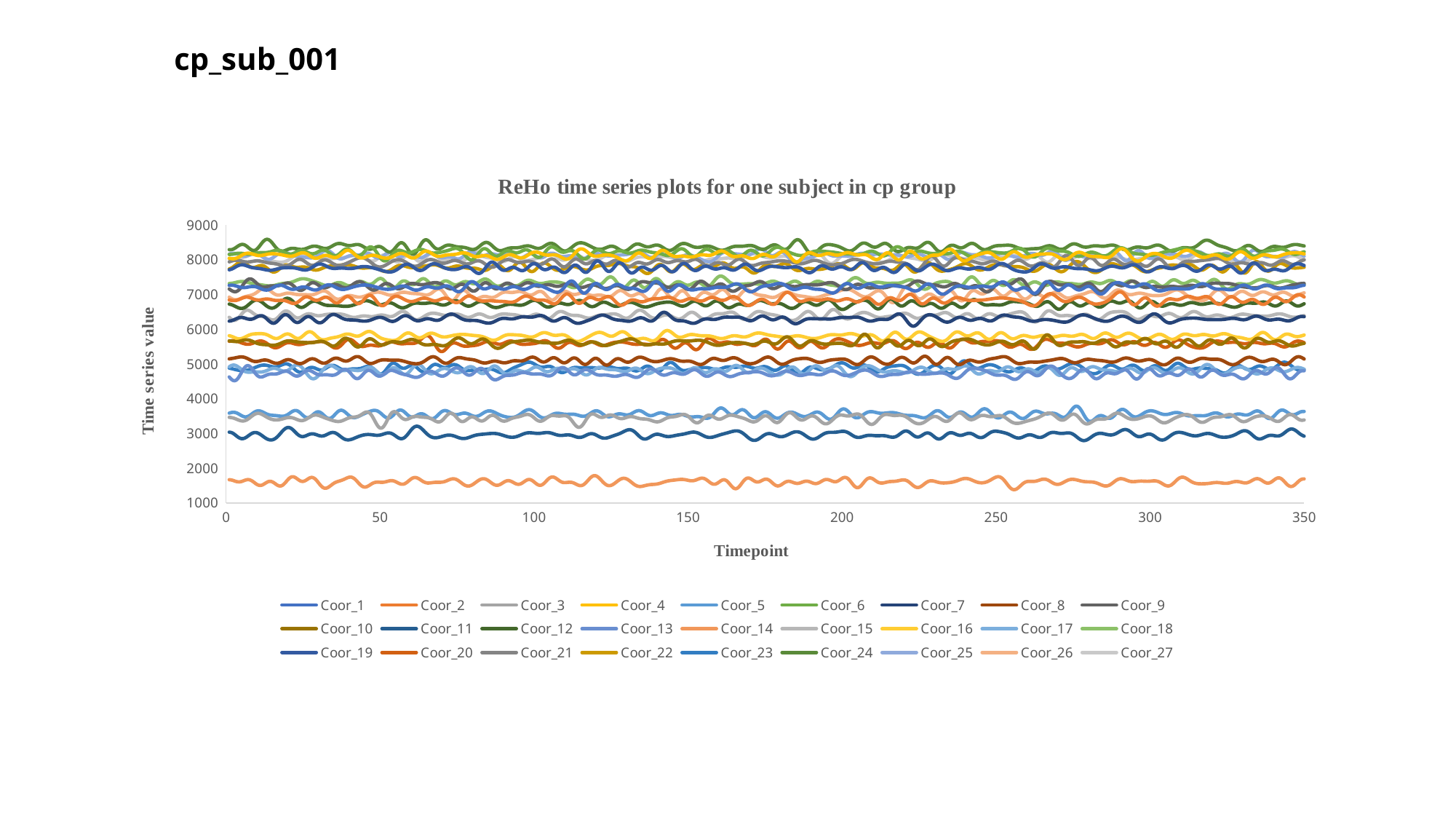

cp_sub_001
### Chart: ReHo time series plots for one subject in cp group
| Category | Coor_1 | Coor_2 | Coor_3 | Coor_4 | Coor_5 | Coor_6 | Coor_7 | Coor_8 | Coor_9 | Coor_10 | Coor_11 | Coor_12 | Coor_13 | Coor_14 | Coor_15 | Coor_16 | Coor_17 | Coor_18 | Coor_19 | Coor_20 | Coor_21 | Coor_22 | Coor_23 | Coor_24 | Coor_25 | Coor_26 | Coor_27 |
|---|---|---|---|---|---|---|---|---|---|---|---|---|---|---|---|---|---|---|---|---|---|---|---|---|---|---|---|

## Slide 4
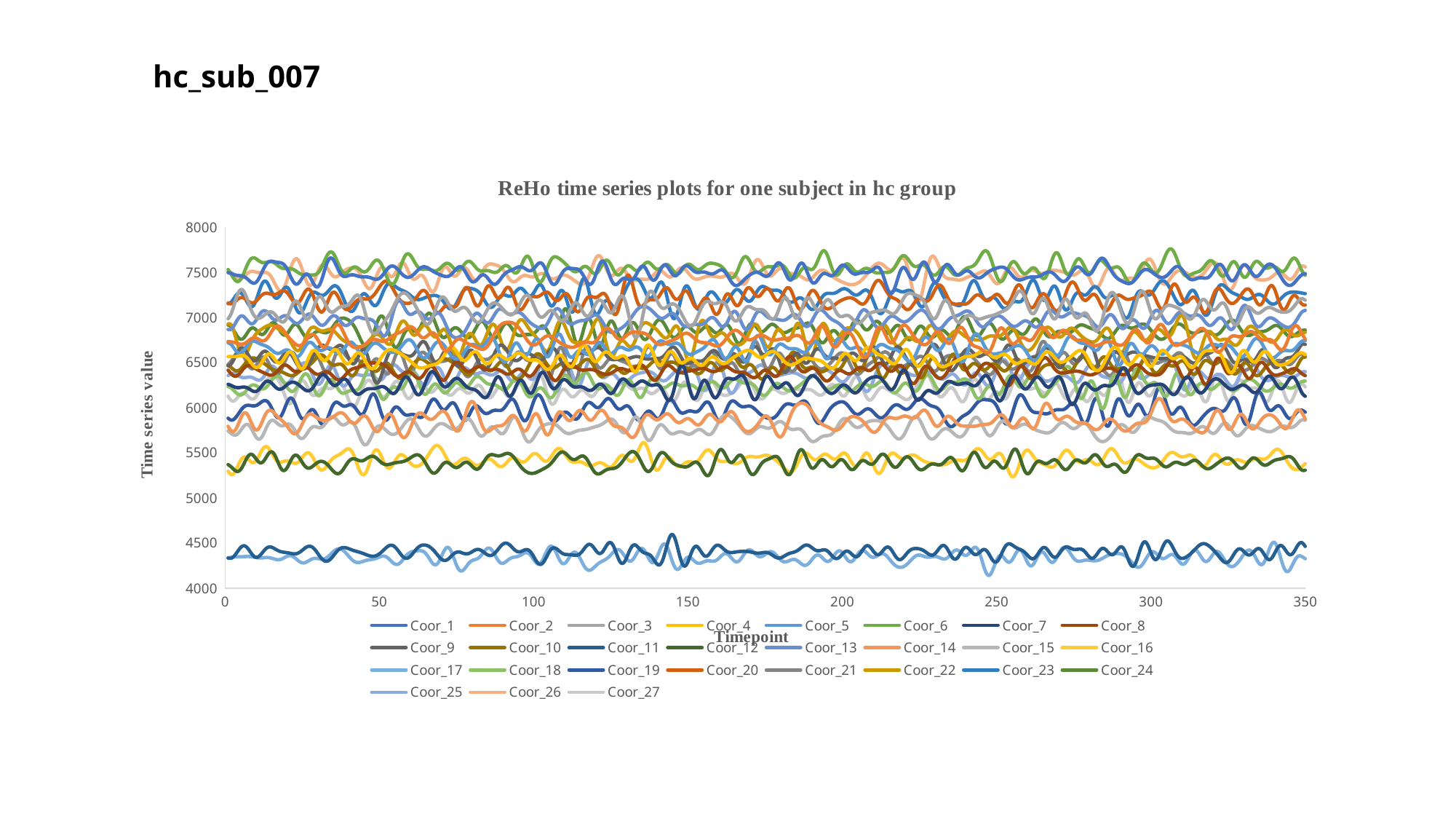

hc_sub_007
### Chart: ReHo time series plots for one subject in hc group
| Category | Coor_1 | Coor_2 | Coor_3 | Coor_4 | Coor_5 | Coor_6 | Coor_7 | Coor_8 | Coor_9 | Coor_10 | Coor_11 | Coor_12 | Coor_13 | Coor_14 | Coor_15 | Coor_16 | Coor_17 | Coor_18 | Coor_19 | Coor_20 | Coor_21 | Coor_22 | Coor_23 | Coor_24 | Coor_25 | Coor_26 | Coor_27 |
|---|---|---|---|---|---|---|---|---|---|---|---|---|---|---|---|---|---|---|---|---|---|---|---|---|---|---|---|

## Slide 5
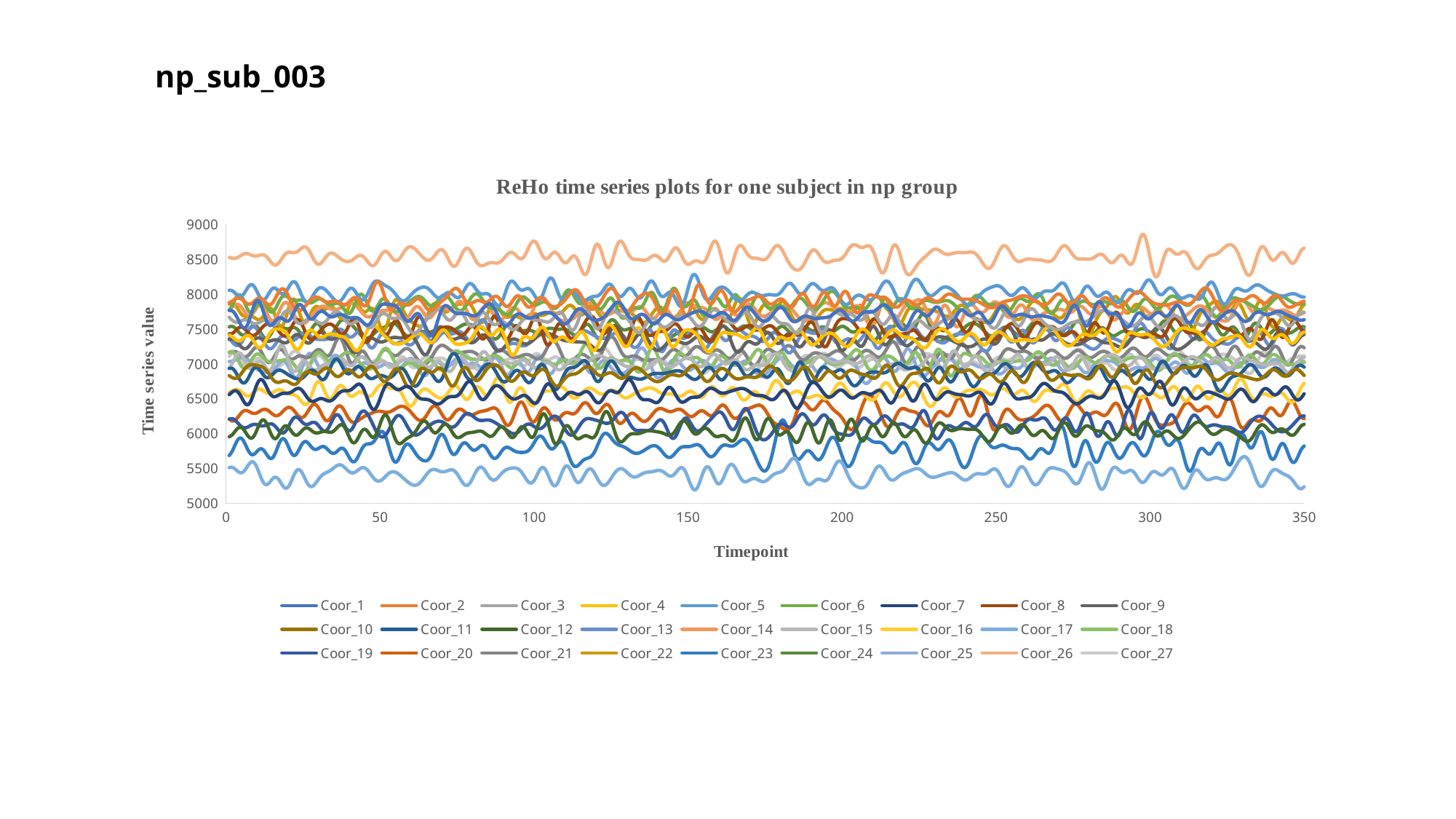

np_sub_003
### Chart: ReHo time series plots for one subject in np group
| Category | Coor_1 | Coor_2 | Coor_3 | Coor_4 | Coor_5 | Coor_6 | Coor_7 | Coor_8 | Coor_9 | Coor_10 | Coor_11 | Coor_12 | Coor_13 | Coor_14 | Coor_15 | Coor_16 | Coor_17 | Coor_18 | Coor_19 | Coor_20 | Coor_21 | Coor_22 | Coor_23 | Coor_24 | Coor_25 | Coor_26 | Coor_27 |
|---|---|---|---|---|---|---|---|---|---|---|---|---|---|---|---|---|---|---|---|---|---|---|---|---|---|---|---|
