# Supplementary material for: Neural functions vary by return-to-sport status in participants with anterior cruciate ligament reconstruction: a retrospective cohort study using sub-bands of resting-state functional magnetic resonance
Source: Front Hum Neurosci. 2024 Nov 1;18:1457823. doi: 10.3389/fnhum.2024.1457823 (PMC11564169; doi:10.3389/fnhum.2024.1457823)
Supplement: Supplementary file 6 [file Table_1.DOCX]

TableS1: Brain regions showing significant differences in SFB, Slow4, and Slow5 among the three groups(GRF correction, voxel p < 0.05, cluster p < 0.05).

| Frequency | Brain regions | Cluster size (voxel size) | MNI coordinates | | | *F* value |
| --- | --- | --- | --- | --- | --- | --- |
|  |  |  | X | Y | Z |  |
| SFB |  |  |  |  |  |  |
|  | Putamen_R (aal) | 95 | 30 | -6 | 0 | 9.0714 |
|  | Putamen_L (aal) | 97 | -18 | 9 | 18 | 19.7646 |
| Slow4 |  |  |  |  |  |  |
|  | Putamen_L (aal) | 59 | -21 | 12 | -6 | 13.2054 |
|  | Precuneus_R(aal) | 58 | 12 | -51 | 69 | 10.4419 |
| Slow5 |  |  |  |  |  |  |
|  | Lingual_R (aal) | 57 | 9 | -51 | 0 | 7.3036 |
|  | Caudate_R (aal) | 66 | 18 | -3 | 9 | 11.2306 |

Note: ALFF, amplitude of low-frequency fluctuations; CP, coper; NP, non-coper; HC, Healthy Controls; SFB, Standard Frequency Band; aal, Anatomical Automatic Labeling; MNI, Montreal Neurological Institute

Table S2 Regions showing significantly differences in ReHo in SFB, Slow4, and Slow5 among three groups (GRF correction, voxel p < 0.05, cluster p < 0.05)

| Contrast | Brain regions | Cluster size (voxel) | MNI coordinates | | | F-value |
| --- | --- | --- | --- | --- | --- | --- |
|  |  |  | X | Y | Z |  |
| SFB | Putamen_L | 254 | -21 | 12 | -6 | 22.3237 |
| Slow5 | Cerebelum_8_R | 204 | 18 | -66 | -45 | 9.5455 |
|  | Putamen_L | 320 | -21 | 12 | -6 | 16.3735 |
|  | Caudate_R (aal) | 292 | 18 | 15 | 21 | 10.1822 |
|  | Cingulum_Mid_R | 268 | 12 | 24 | 36 | 12.7838 |

Note: ALFF, amplitude of low-frequency fluctuations; CP, coper; NP, non-coper; HC, Healthy Controls; SFB, Standard Frequency Band; aal, Anatomical Automatic Labeling; MNI, Montreal Neurological Institute
